# Editorial: Advances and trends in microbial production of biopolymers and their building blocks

**DOI:** 10.3389/fbioe.2022.1025797

**Published:** 2022-09-27

**Authors:** Xinjun Feng, Xinglin Jiang, Guang Zhao

**Affiliations:** ^1^ CAS Key Laboratory of Biobased Materials, Qingdao Institute of Bioenergy and Bioprocess Technology, Chinese Academy of Sciences, Qingdao, China; ^2^ The Novo Nordisk Foundation Center for Biosustainability, Technical University of Denmark, Kongens Lyngby, Denmark; ^3^ State Key Laboratory of Microbial Technology, Shandong University, Jinan, China

**Keywords:** biopolymer, polyhydroxyalkanoates, polysaccharide, poly (lactic acid), succinic acid, 3-hydroxypropionic acid, microbial technology, carbon emission

Petroleum-based polymers have played an important role in human daily life, but they also cause serious environmental pollution due to their low biodegradability. In contrast, biopolymers have attracted unprecedented attentions with advantage of life cycle carbon emission reduction under the global achievement of “carbon peak” and “carbon neutrality”. However, some shortcomings of biopolymes remain and restrict their popular application, such as high cost, low productivity, and poor material properties.

This special issue consists of six original papers, one brief research report, two specialized review papers and one mini review article covering workhorse strains, cheap raw materials utilization, enzyme screening, monomer biosynthesis and bio-process optimization, which showcase current progress on biopolymers and aim to provide possible solutions to the existing problems.

Polyhydroxyalkanoates (PHAs) are one of the most popular biopolymers that exhibit similar mechanical properties to petrochemical-derived polymers with the added benefit of biodegradability and biocompatibility (Rekhi et al., 2022). To date, over 150 types of PHAs have been produced in the form of homopolymers, random and block copolymers, thus supporting a wide range of applications. Gao et al., systematically reviewed the recent advances in microbial synthesis of PHAs, including chassis engineering, substrate utilization, PHA synthase modification, and scale-up biomanufacturing, which will be of special interest to the researchers in the field of PHA biosynthesis.

The microbial hosts are crucial to desired biopolymer production. More than 70 genera of microbes have been identified and/or engineered for PHAs production (Alcântara et al., 2020), such as *Ralstonia eutropha*, *Pseudomonas*, *Escherichia coli*, etc. The choice of microbiological strains directly impacts the chemical composition, structure and properties of the final polymers, as well as the substrate used. In recent years, some bacteria with particular characteristics have been proved to be economical and effective hosts. *Halomonas bluephagenesis*, a halophilic bacteria, can accumulate poly-3-hydroxybutyrate (PHB) up to more than 80% of the cell dry weight in an open unsterile process (Tan et al., 2011). *Rhodopseudomonas palustris*, a photosynthetic bacteria, has the ability to convert various carbon sources, especially CO_2_, into valuable chemicals and biopolymers. A review by Li et al. provided a comprehensive overview of the advantages, the challenges and possible solutions of *R. palustris* as a powerful microbial cell factory, specifically discussed its applications in production of PHAs, polysaccharides and isoprenoids monomers.

PHA synthases polymerize various hydroxyacyl-CoAs into different PHA polymers. However, PHA synthases prefer 3-hydroxyacyl-CoAs rather than 2-hydroxyacyl-CoAs, which makes it very difficult to synthesize polylactic acid (PLA) directly in microbial cells. To screen robust PHA synthase for PLA homopolymer biosynthesis, Shi et al. evaluated the class I PHA synthase from *Chromobacterium* sp. USM2 in engineered *E. coli* using glucose as carbon source, and demonstrated that it is feasible to catalyze the polymerization of lactyl-CoA with better performance in PLA production than that of the evolved class II PHA synthase PhaC1_Ps6-19_. This work proved that class I PHA synthase has catalytic ability to 2-hydroxyacyl-CoAs.

As the cost of the substrate can account for up to 50% of the overall production cost (Urtuvia et al., 2014), various renewable and inexpensive carbon sources including lignocellulosic biomass hydrolysates, sugarcane molasses, crude glycerol, have been evaluated for PHAs production. Szacherska et al. explores to use short- and medium-chain fatty acids (SMCFAs) as raw materials for PHA production in three individual *Pseudomonas* strains. This study demonstrated that high PHA productivity can be obtained in *Pseudomonas* sp. Gl06 cultivation under nitrogen limitation conditions with cheap SMCFA-rich stream from a cheese production line, and the extracted polymers possess better properties with lower melting point and degradation temperature.

Moreover, many bio-monomers can be utilized for polyesters production directly through chemical polymerization. Succinic acid is one of building blocks of the commercial polyester poly (butylene succinate) (PBS) with wide temperature window for thermoplastic processing. Liu et al. summarized different succinic acid biosynthetic pathways, concentrating on the key enzymes and metabolic engineering approaches, and future perspectives were also proposed. 3-Hydroxypropionic acid (3-HP) is a versatile platform compound which can be polymerized into polymers. Liu et al. engineered a 3HP biosynthetic pathway from glucose with glycerol as intermediate in *Klebsiella pneumoniae*, and improved 3-HP production by CRISPR interference. 2-fluoro-3-hydroxypropionic acid was firstly biosynthesized by Liu et al., which is expected to obtain fluorine-containing materials such as poly (2-fluoro-3-hydroxypropionic acid) with better properties. Liu et al. investigated the biosynthesis of lactate in *E. coli* and proved that lysine acetylation of lactate dehydrogenase can regulate lactate synthesis effectively. In another study, Zhang et al. improved the bioconversion of phenylpyruvate to l-phenyllactate, whose homopolymer exhibits high-ultraviolet-absorbing properties, by introducing a NADH-dependent l-lactate dehydrogenase mutant into *Pichia pastoris* coupling with a NADH regeneration system.

As an important natural biopolymer, polysaccharides have been widely used in the food, pharmacy, cosmetics, and chemical industries, and microbial polysaccharides in particular offer many advantages compared to plant-derived polysaccharides (Chaabouni et al., 2014). Xu et al., presented the systematic optimization of fermentation process of exopolysaccharide using *Candida glabrata* mutant, resulting in significantly improved production of exopolysaccharide that shows great industrial application potential.

Finally, we sincerely thank all the submitting authors for considering to share their updated research outcomes and opinions in this special issue, the reviewers for their time and constructive comments to improve the manuscripts, also the editors Dr. Xiao-Jun Ji and Dr. Ka Yu Cheng for their kind support. We hope that readers find these articles in this special issue interesting and useful for their own research.
